# Congenital scoliosis: an up-to-date

**Published:** 2015

**Authors:** G Burnei, S Gavriliu, C Vlad, I Georgescu, RA Ghita, C Dughilă, EM Japie, A Onilă

**Affiliations:** * ”M. S. Curie” Clinical Emergency Hospital for Children, Bucharest, Romania; ** "Carol Davila” University of Medicine and Pharmacy, Bucharest, Romania; *** "Regina Maria” Private Medical Network, Baneasa Hospital, Bucharest, Romania

**Keywords:** congenital scoliosis, spinal imbalance, progressive potential, growth, spinal implants

## Abstract

Congenital scoliosis represents a spinal malformation due to defects of formation, segmentation or mixed ones. It is characterized by a longitudinal and rotational imbalance.

54 patients were analyzed and 39 out of them were operated by various approaches with anterior and posterior instrumentations during 2000 and 2012. The impossibility to appoint some patients encountered in the daily practice into the known classifications, allowed us to purpose two categories of congenital scoliosis related to the predominance of spinal deviances in the coronal and transversal views.

No certain etiology of congenital scoliosis has been identified until today. The susceptibility of some polygenic defects is obvious due to the presence of a sum of defects associated to most of the congenital scoliosis cases and the rarity of the presence of a unique defect.

The diagnosis requires a thorough clinical and imaging examination in order to establish an individualized therapeutic strategy.

The treatment of congenital scoliosis is different from the adolescent idiopathic one. Therapeutic criteria are significantly different. It is essential to assess the difference in growth of the concavity related to the convexity when choosing a particular procedure. The magnitude of the curve and the progressive rate are fundamental issues to the surgeon

## Introduction

Our experience based on 39 cases of congenital scoliosis operated by various approaches and both anterior or posterior instrumentations allowed us to mention the cumulated notions with those present in literature.

Congenital scoliosis is a malformation characterized by a longitudinal and rotational imbalance.

**Scoliosis with imbalance in the longitudinal growth** is produced by defects of formation, segmentation or mixed ones.

**Scoliosis with rotational imbalance** is mainly characterized by the vertebral rotation related to the curve in the coronal plan. These types of scoliosis are secondary to a congenital malformation, either vertebral or pelvic, which induce as the main manifestation the vertebral rotation by means of traction, pushing or mixed action. Usually, the scoliosis and the vertebral rotation are not present at birth. During growth and development, the first presence is the vertebral rotation accompanied by walking impairment and next by the scoliotic curve.

## Classification

The first classification of congenital scoliosis based on X-rays imaging was described by Winter in 1968 [**1**]. Kawakami (2009) reclassified the vertebral malformations depending on the presence or absence of normal formation based on a 3D-CT study [**2**,**3**]. The purpose of these classifications is to understand the embryology, etiology, prognostic and to choose the right therapeutic strategy.

The grouping of the cases presented in literature and the impossibility to appoint some patients encountered in the daily practice into the known classifications, allowed us to purpose a further classification containing 2 large categories related to the predominance of spinal deviances in the coronal and transversal plans: scolioses with longitudinal imbalance and with rotational imbalance.

**Congenital scoliosis with longitudinal imbalance**, mainly with deviation in the coronal plan in comparison with vertebral rotation, may be due to defects of formation with the presence of: trapezoidal vertebra, hemivertebra or vertebral hemibody. Hemivertebra depending on segmentation may be fully segmented, hemisegmented (partially segmented) or unsegmented. There may be congenital malformations characterized by the presence of more than one hemivertebra disposed in the following manner: *adjacent* (successive) - 2-3 hemivertebrae disposed unilaterally inducing a short arch scoliosis, being noticed at birth and having a high rate of evolution, *unilateral alternant* (intermittent) - 2-3 hemivertebrae placed unilaterally leading to a long arch scoliosis and a unique curve and *bilateral alternant* which themselves may be:

• *compensated* - 2 symmetric hemivertebrae in a 4-5 vertebral segment inducing an equilibrated spinal deformity not requiring surgery;

• *uncompensated* - if the hemivertebrae are disposed on a distance of more than 6 vertebrae leading to a double congenital scoliosis.

Due to defects of segmentation, congenital scolioses are characterized by a unilateral defect: longitudinal bar or a bilateral defect: vertebral block.

The third possibility is represented by mixed anomalies where we may find the next malformations:

• hemivertebra and a bar on the opposite spinal side;

• hemivertebra, vertebral block and longitudinal bar.

**Congenital scoliosis with rotational imbalance** is mainly characterized by vertebral rotation compared to the deviation in the coronal plan and they may be due to an effect of:

• spinal traction - osseous bridges with congenital transverso-sacrate synostosis;

• spinal pushing - mega-apophysis of the L5 transverse process;

• mixed (traction and pushing) - sacral agenesis with pelvic malposition.

## Etiopathogeny

Up until today, there has been no certain etiology of congenital scoliosis. The susceptibility of some genetic defects, polygenic ones, is obvious due to the presence of a sum of defects associated with most of the congenital scolioses and the rarity of the presence of a unique defect.

There are a series of factors that may favor the production of these congenital anomalies. The exposure to carbon monoxide in the formation period of the somites may lead to these defects. Another predisposing factor may be fetal hypoxia either of maternal conditions, fetal or placental [**4**]. There are also some other inducing factors described like gestational diabetes, intake of antiepileptics, prolonged febrile states of the pregnant women or exposure of the fetus to temperatures higher than normal.

## Clinics

**Fig. 1:**
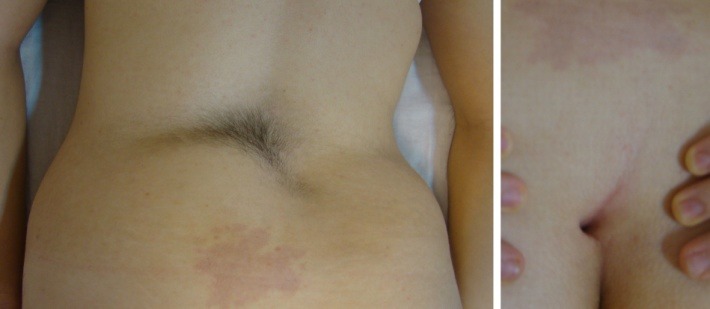
Hypertrichosis and dermal sinus with an underlying congenital scoliosis

**Fig. 2:**
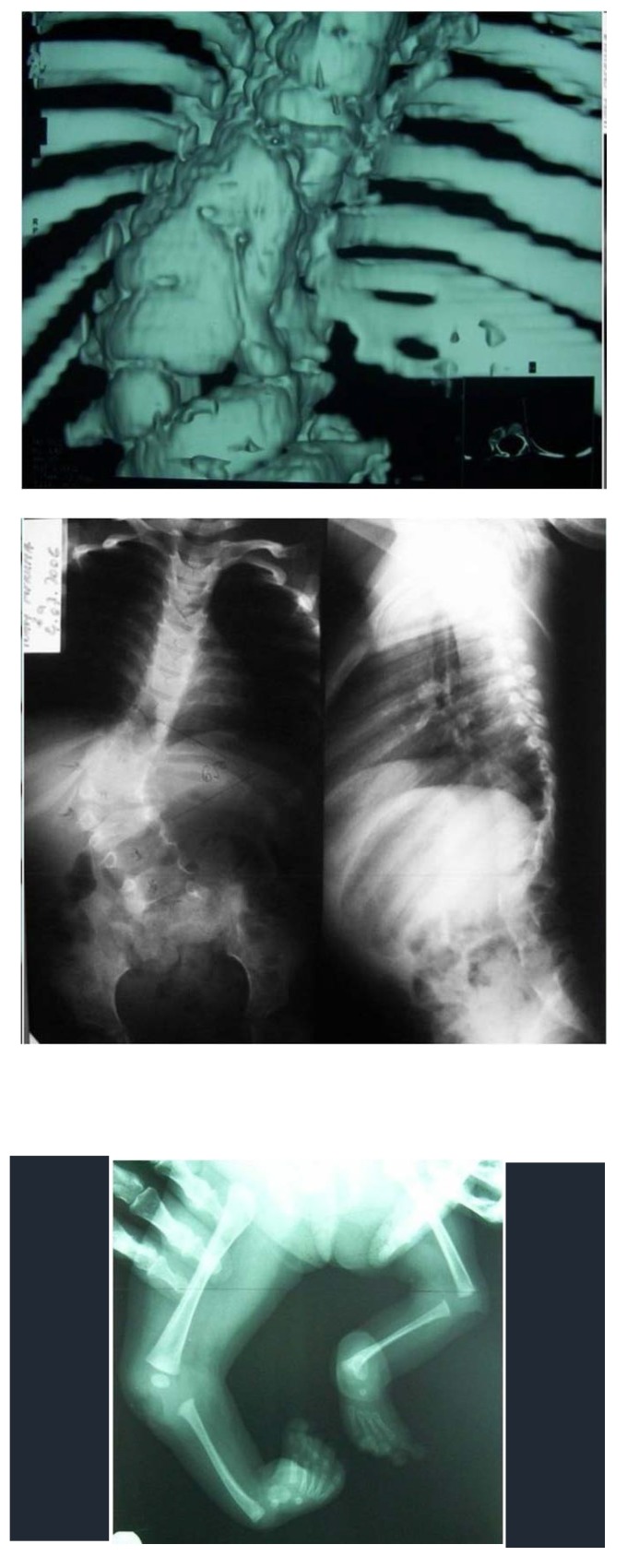
Severe congenital scoliosis in a 4-year-old child with bilateral hemimelia (absence of the tibia). The spinal malformation consisted in bilateral vertebral block and alternant, intermittent hemivertebrae

Height and weight, body imbalance, teguments (**Fig. 1**), thoracic deformities [**5**], associated congenital malformations (**Fig. 2**), motor and sensitive disorders [**6**] should all be checked.

**Associated anomalies**

• *Neurologic malformations*

Congenital scolioses are associated in 35% of the patients with other neurologic malformations related to the nervous system and its coating. The most frequently encountered are diastematomyelia, Chiari’s malformation, intradural lipoma and tethered cord.

• *Congenital heart malformations*

Congenital heart malformations are present in up to 25% of the patients with congenital scoliosis. Severe anomalies like Fallot tetralogy or the transposition of the great vessels require surgery prior to a spinal surgical approach [**7**].

• *Urologic anomalies*

**Fig. 3:**
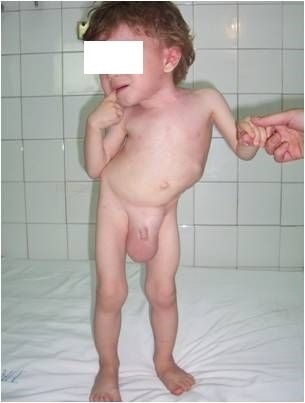
Giant inguinal hernia in a child with congenital scoliosis

Urologic anomalies are encountered in 20% of the cases. These anomalies associated to congenital scoliosis are horseshoe kidney, vesicoureteral reflux or hypospadias. We may encounter inguinal hernia, which is usually of great dimensions, needing surgery, too (**Fig. 3**).

• *Musculoskeletal anomalies*

These malformations, clinically and imagistically detected, are usually treated after scoliosis surgery. They include Sprengel’s disease, Klippel-Feil syndrome [**8**], congenital femoral hypoplasia or acetabular dysplasia.

## Natural history

**Fig. 4:**
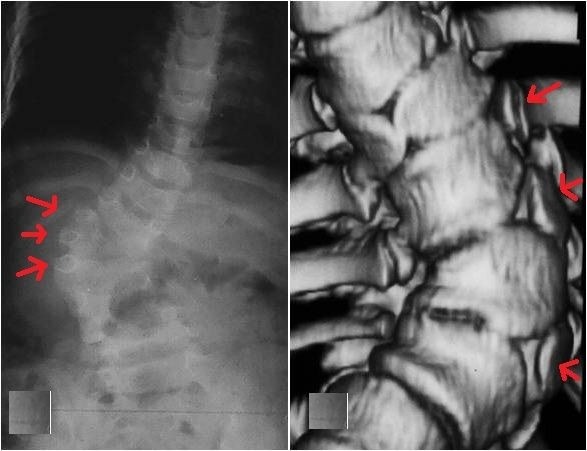
Congenital scoliosis due to 3 successive fully segmented hemivertebrae (left) and to 3 intermittent hemivertebrae (right)

It is very important for the practitioner to establish the progressive potential of the scoliotic curve after identifying the type and localization of the deformity. The curve’s progression is induced by an unequal accelerated growth on the side with a hemivertebra or on the opposite one to the presence of a longitudinal bar [**9**]. The progression prognosis is evaluated by intervertebral disks check by X-rays exam. When these are well individualized, the growth plates of the vertebrae are present and on the side with a hemivertebra, the growth is more rapid. That is why a fully segmented vertebra with well-defined intervertebral disks has a more progressive potential in the deforming of the spine in comparison with an unsegmented vertebra. A much higher rate of progression is noticed by the presence of 2-3 successive or intermittent fully segmented hemivertebrae (**Fig. 4**) [**10**]. 

The progression rate depends on the type of anomaly, the patient’s age and the place of the curve. The highest progression potential is related to scolioses due to mixed defects with fully segmented hemivertebrae on one side and a unilateral bar on the other [**11**]. The progression rate is of about 10 degrees per year. The curve progresses rapidly in the first 5 years and next during the age between 11 and 14 years. These are the growth spurt periods of the vertebrae. The spine involving congenital anomalies may be equilibrated both clinically and on X-rays exams when 2 hemivertebrae are disposed symmetrically on both sides of the spine on short segments of no more than 4-5 vertebrae. If the vertebra is a butterfly one, the spine is balanced in the frontal plan, but there is an initially mild kyphosis present. If there are more than one hemivertebrae, successive or intercalary, the kyphosis will get severe. The spine may be imbalanced in the presence of fully segmented adjacent or intermittent hemivertebrae.

**Fig. 5:**
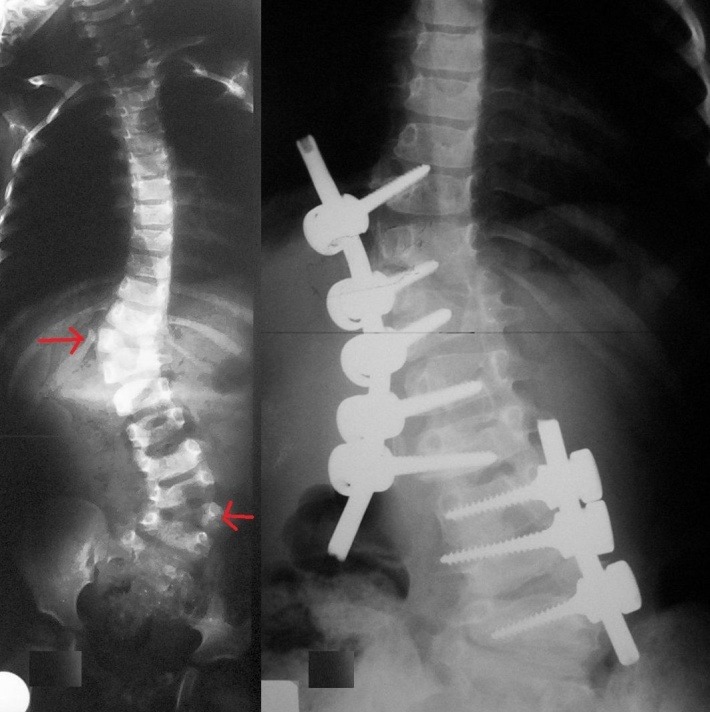
Double curved congenital scoliosis due to 2 hemivertebrae disposed bilaterally, with a segment of 5 normal vertebrae in between

The anomalies situated in the cervico-thoracic or lumbosacral area produce deformities more obvious than those situated elsewhere. In the cervical area, most deformities are due to segmentation defects [**12**]. The presence of 2 hemivertebrae placed on a distance of more than 5 normal vertebrae, but situated on both sides of the spine induce a congenital double thoracolumbar scoliosis, which may be approached in an-one stage surgery or in separate procedures (**Fig. 5**).

Morbidity and early mortality are primary due to chronic pulmonary lesions and chronic heart disease [**13**].

## Imaging

• *Plain X-rays*, frontal and lateral, represent the usual investigation used to diagnose a congenital scoliosis and to evaluate the spontaneous evolution, during preoperative bracing and after surgery. The evaluation aims to measure the curves’ angle by Cobb’s or Fergusson’s method and to spatially appreciate the deformity. Repetitive exams allow the quantification of the curve in order to compare the results of different treatment methods. 

• *Computed Tomography (CT-scan) and 3D-CT* are indicated in the evaluation of complex anomalies before surgery in order to identify anatomical particularities and to localize bony malformations in the operative area. The presence of these malformations may be an unpleasant surprise for the surgeon leading to severe complications. CT and 3D-CT to analyze the thorax and the lung highlights the thoracic wall deformities: rib synostoses, rib hypoplasia or agenesis, intracanalar protrusions through the radicular foramina, etc. 3D-CT evaluates the thoracic insufficiency syndrome due to congenital scoliosis and thoracic wall malformations.

• *Magnetic resonance imaging (MRI)* is used as a substitute to the abandoned myelography and it offers much better information about occult spinal malformations. These are present in 30% of the patients with congenital malformations.

## Treatment

The presence of a scoliotic deformity at birth is a sign of worse prognosis and it requires treatment starting with the first days of life. Not all scolioses need bracing or surgery. 25% of them present a low progression rate or compensating defects of formation. These deformities have to be periodically evaluated and usually do not require surgery.

About 75% of congenital scolioses require surgery. Surgery is indicated at the age of 1-4 years.

The essential criteria to choose the right moment of surgery is the magnitude of the scoliotic curve. Evaluation is performed by measuring the Cobb’s angle. Up to 40°, the patient is periodically carefully monitored, at every 4-6 months. Above 40°, surgery is required. The presence of a respiratory disorder associated to some congenital malformations endangers the patient and imposes a more careful supervision and surgery as soon as possible. Congenital scoliosis with more than one fully segmented, successive hemivertebrae and severe deformities of the rib cage with thoracic insufficiency syndrome may be operated at the age of 8-12 months, even if Cobb’s angle is less than 40°.

**In situ fusion**

This procedure, even if being a safe technique, presents certain indications because correction possibilities are limited. It is indicated in progressive scoliosis presenting with a minimal deformity at surgery time, no more than 25°, with a limited area of no more than 5 vertebrae. It may be regarded as a prophylactic act in cases of high rate progression scoliosis with a fully segmented hemivertebra. This kind of arthrodesis insignificantly limits the growth in length of the spinal column and it may be used as an elective procedure in children with age ranging 1 to 4 years.

Present deformities correct slowly and the result is efficient if the growth potential is properly assessed by a CT-scan or MRI. *In situ* fusion may be performed by an open anterior approach, by thoracoscopy or by an open posterior approach through the pedicles. Usually, the surgeon chooses one of these options depending on its experience and the deformity’s location.

**Convex hemiepiphysiodesis**

The elective indication is congenital scoliosis due to defect of formation with the presence of a hemivertebra [**14**]. During surgery, the correction of the curve is partially obtained, the remaining correction being achieved slowly in time because of the ablation of the intervertebral disks on the convex side. 

Best results are achieved if the procedure is performed in a child with age ranging 1 to 4 years. All over, in the long term, same as for *in situ* fusion, the correction limit is up to 20-25°. Argues about its indication are present in literature due to the fact that expected results were not obtained. Our opinion is that we may obtain good results if the curve is less than 30°, if it is associated to posterior fusion, the progressive rate before surgery being constantly of 8-10° per year and the malformation being a fully segmented vertebra. The approach is via a thoracotomy or an abdomino-thoracotomy on the side of the convexity depending on the level of the malformation.

**Excision of the hemivertebra**

It is the best treatment method in comparison to in situ fusion and hemiepiphysiodesis. Maximal efficiency is obtained if performed at the age of 1-4 years, when the hemivertebra has a thoracic, lumbar or lumbo-sacral position and there is an imbalance of the trunk. Excision may be performed by an anterior or a posterior approach [**15**]. A posterior approach is indicated in case of an isolated resection. A posterior and anterior simultaneous approach allows a complete excision of the adjacent disks of the hemivertebra by a circumferential exposure. This allows total visibility when excising the hemibody and the pedicle. This kind of approach requires the reposition of the patient during surgery. We prefer a successive approach during the same surgical procedure by rotating the patient in the same sterile field, next instrumenting the spine after the hemivertebral excision. During 1998 and 2006 we practiced 23 procedures with a medium correction of 64% (an average of 41° preoperatively to an average of 16° postoperatively) [**16**]. The evolution in time of the deformity has been of about 3-4° per year, requiring a conversion of the anterior instrumentation to a posterior one at puberty in order to stabilize the spine.

Posterior excision of the hemivertebra ensures very good results. This method is best for a hemivertebra located in the thoracolumbar junction and is accompanied by kyphosis [**17**,**18**].

**Fig. 6:**
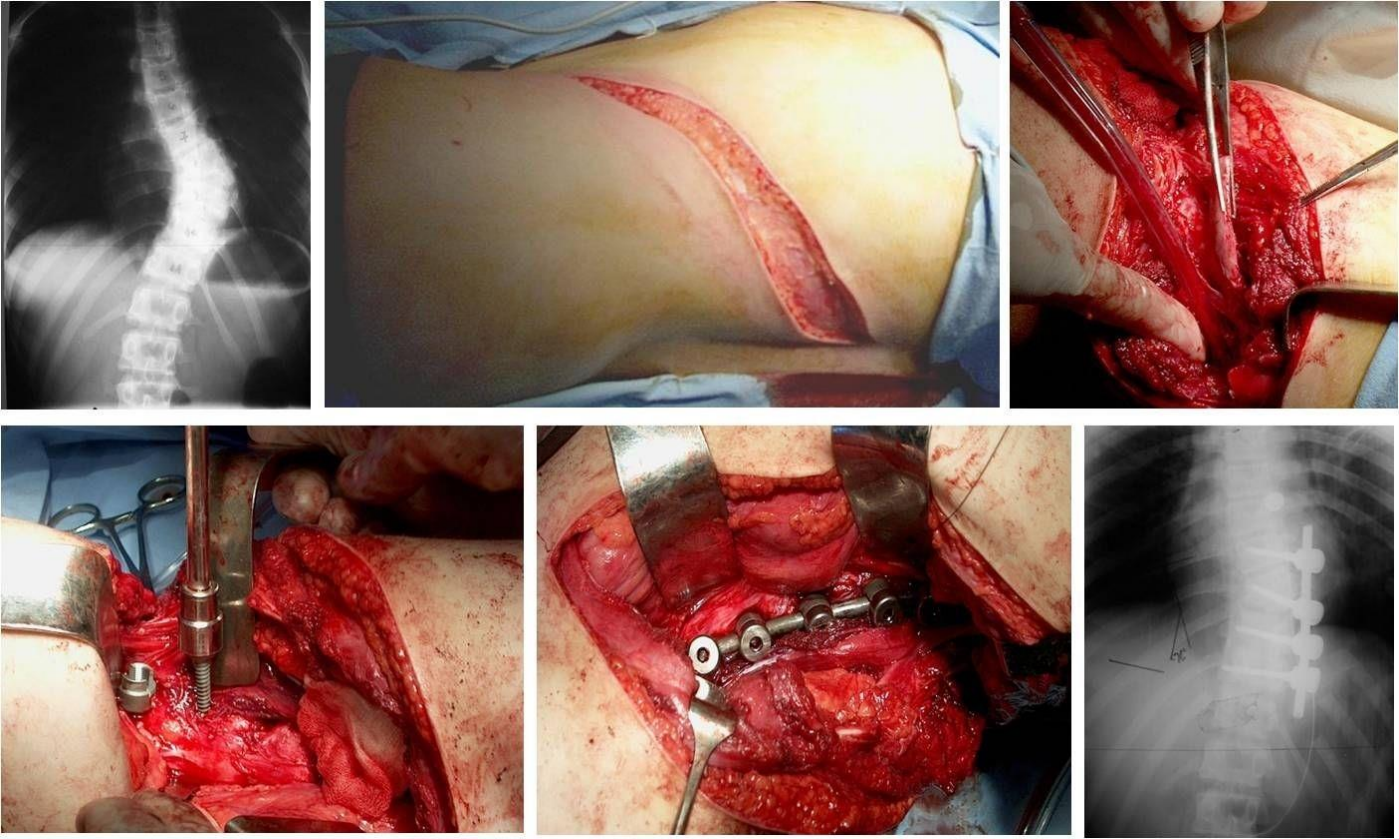
Mirbaha approach and somatic synthesis in a congenital scoliosis due to a fully segmented T9 hemivertebra

Our experience is based on a Mirbaha approach (extrapleural, retroperitoneal) in order to excise T11-L5 hemivertebrae, too. This approach allowed somatic instrumentation. T11 and T12 hemivertebrae were excised completely, anterior and posterior parts of the hemivertebra, by this single approach (**Fig. 6**). 

L1-L5 hemivertebrae were excised by a double approach and the next somatic instrumentation was performed. T9 and T10 were excised both anterior and posterior after the resection of the 10th and 11th rib.

In 2002, Ruf and Harms described the resection of hemivertebra by a posterior approach followed by fixation with transpedicular screws [**19**].

The excision of the hemivertebra is recommended if the curve progresses rapidly. It becomes an emergency in the presence of spinal canal stenosis or disk hernia, as a measure of decompression. The approach is variable depending on the site of the hemivertebra: Transthoracic for T4-T11, Hodgson (transpleural, retroperitoneal for T9-L5), Burnei (transthoracic, retropleural for T2-T11) and Mirbaha (extrapleural, retroperitoneal for T11-L5).

## The role of spinal instrumentation and vertebral fusion

The correction of the spinal deformity may be partially or complete. Partial correction is performed during surgery by hemivertebral/ vertebral resection or postoperatively by in situ fusion, convex hemiepiphysiodesis or bracing. Curves of more than 20° cannot be corrected without spinal instrumentation. Initiated by Hall in 1981, thanks to technical progress, the spinal instrumentation has been a revival in the use of small sized titanium made implants allowing a full postoperative evaluation [**20**].

Different types of spinal implants are now available to correct the deformity as much as possible and allow the growth of the spine and thoracic cage. Skaggs classifies the devices of spinal distraction and thoracic expansion in systems based on distraction, compression and modulated growth [**21**].

**Growing rods**

The first to initiate this concept was Harrington in 1960. The promoter of this method is Akbarnia who improved the distraction device by using tandem connectors on 2 rods with distraction possibilities. He succeeded in correcting the angle from 82° to 38° in cases of early onset scoliosis and ensured a 1.2 cm/ year growth of the spine [**22**].

This method is a fusionless curve correcting one. Growing rods have become implants suitable in congenital scoliosis with large curves with normal disks above and below the malformation or the curve’s apex and with flexibility of the upper and lower segments of the spine. Growing rods are more suitable due to the fact that children younger than 5 years treated by thoracic fusion developed important respiratory problems finally leading to respiratory insufficiency. Data regarding physiopathology, growth and development of the spine and thoracic organs up to 5 years showed that the height and volume of the vertebra are of about 70% of an adult. That is why congenital scoliosis is associated with a diminished trunk height and, as a consequence, a shorter stature. Arthrodesis in these children will lead to a more reduced trunk height and thoracic volume. Nowadays, fusion is avoided in children of less than 10 years of age, just as Harrington predicted.

Growing rods are distracted at every 6 months. Transpedicular screws have to be used with caution in the upper thoracic part in very young children, but if required at least 4 should be used in order to spread the local tension [**23**]. If the established spinal anchoring points prove to be anomalous not allowing the placement of implants, a VEPTR should be used.

**Halo traction**

If the spinal column in congenital scoliosis is very stiff halo traction should be used before surgery. Traction is indicated even in some cases with neurological problems. It is a gravitational traction allowing the patient to sit in bed or walk with a wheelchair or with any other walking device. Gravity will ensure a partial reduction of the curve up to 70% before surgery without any neurological issues. If required, the traction weight will be diminished or even suppressed. 

**VEPTR (Vertical Expandable Prosthetic Titanium Rib)**

**Fig. 7:**
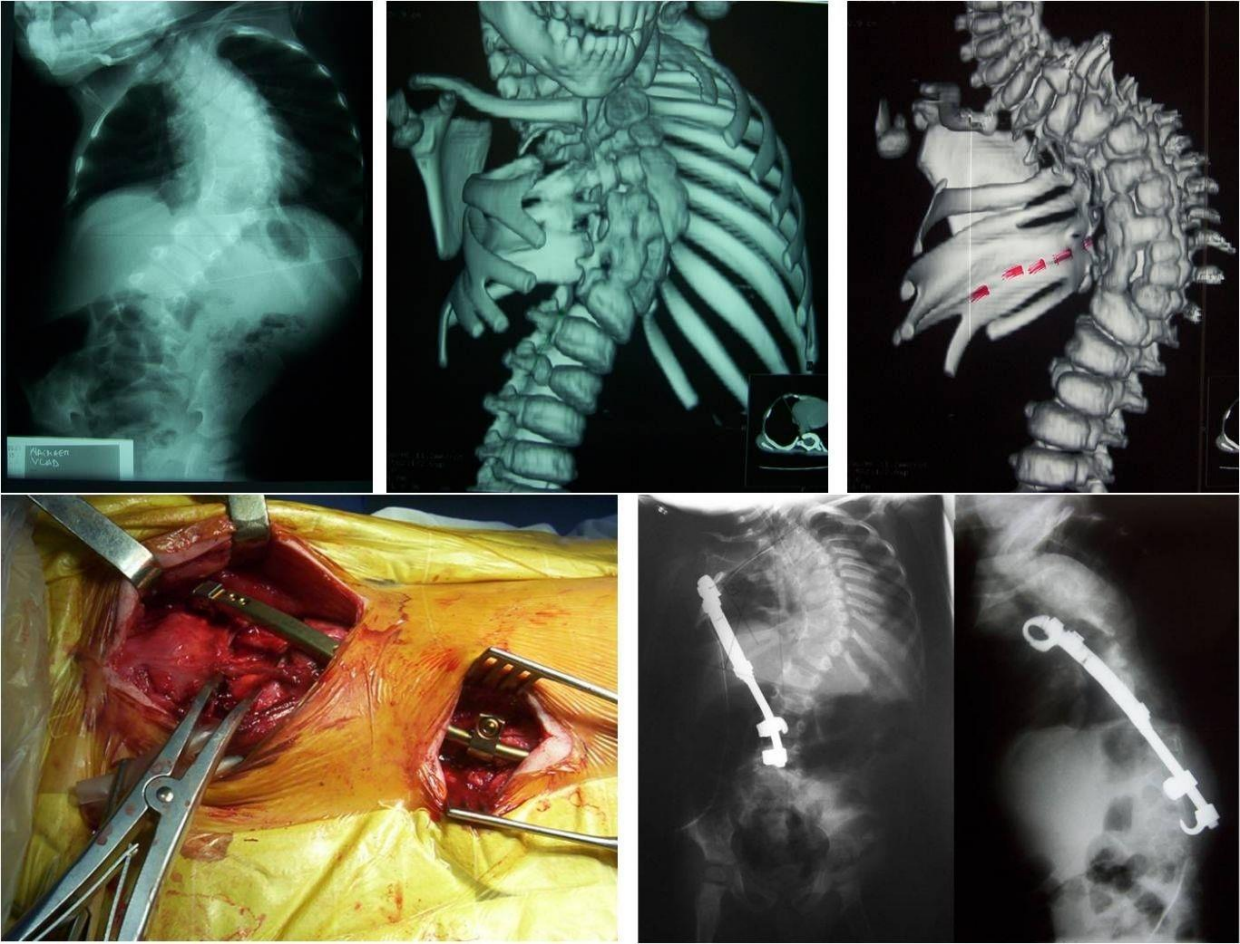
Congenital scoliosis with rib agenesis and rib fusion treated by thoracostomy and VEPTR device

This device ensures a progressive correction of the curve and the expansion of the thorax by a thoracotomy. The elective indication is in cases of scoliosis associated to rib synostoses and thin thorax that induce a defective lung development and evolve to thoracic insufficiency if not treated [**24**]. Thoracic volume may be increased by the use of VEPTR, fixed rib-to-rib or more frequently rib-to-spine. If the deformity is a lumbar one and the pelvis is unbalanced, a device rib-to-ilium is indicated. Expansion thoracoplasty resides in the axial sectioning of the bony synostotic rib plate followed by intra-operative distraction maintained next by the aid of VEPTR (**Fig. 7**).

Contraindications of VEPTR consist in inadequate strength of bone for the attachment of the device, absence of ribs for attachment, inadequate soft tissue for coverage, age of less than 6 months, absent diaphragmatic function, allergy to material, infection at the operative site and age beyond skeletal maturity or spinal canal stenosis. 

In our series, we met a case of congenital scoliosis with spinal stenosis due to the protrusion of the 11th and 12th rib into the canal, which were removed before scoliosis correction [**25**].

This method allows an important correction of the Cobb angle up to 60% and the vital capacity of the lung remains the same or even it increases in some cases. As a rule, the spine will grow and the volume of the hemithorax increases without an improvement of the functional pulmonary volume. The current results showed a benefic result especially in congenital scoliosis associated to chest wall deformities [**26**,**27**]. If needed, the device may be repositioned or converted. The complications in the use of VEPTR are bone erosion, skin breakthrough, infection, post-operative pain, device fracture due to stress fatigue, scapular elevation, brachial plexus palsy [**28**] and medullar lesion, as an exception.

We used a VEPTR device in a vertebra-to vertebra construct in a 1 year and 10 months old patient with Ist-IVth rib agenesis on the concave side of the scoliotic curve [**29**].

**Fig. 8:**
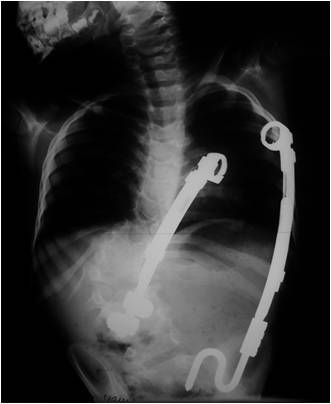
Placement of 2 VEPTR (rib-spine and rib-ilium) in a congenital scoliosis

The thoracoplasty is adequate to the simultaneous treatment of the scoliotic curve, thoracic expansion and chest wall lesions [**5**,**30**]. A proper correction of the thoracic deformity may require the use of 2 or more such devices (**Fig. 8**).

**Guided-growth implants**

Growing rods and VEPTR require periodic minimal invasive procedures for distraction. The use of guided-growth implants like Shilla or a modified Luque trolley presents the advantage of an in situ correction and arthrodesis of the apical site of the deformity. Spinal growth is ensured and guided by the implant below and above the apex of the curve [**31**,**32**]. The guided-growth implants are indicated in early onset, neuro-muscular and syndromic scoliosis in children less than 10 years of age. 

We operated in our clinics a 2 years and 7 months old patient with a double congenital scoliosis due to 2 hemivertebrae disposed bilaterally alternant. The spine presented 4 supernumerary vertebrae, out of which 2 were hemivertebrae, one a fully segmented in the thoracic area and the other hemisegmented in the lumbar area. The other supernumerary vertebrae were disposed as T14 and T15. Surgery consisted in a posterior approach, both hemivertebrae resection and the placement of a guided-growth implant after both apexes of the double congenital scoliosis and T14-T15 was fused. Curve correction was performed *in situ*.

## Principles and strategies in the treatment of congenital scoliosis

**Fig. 9:**
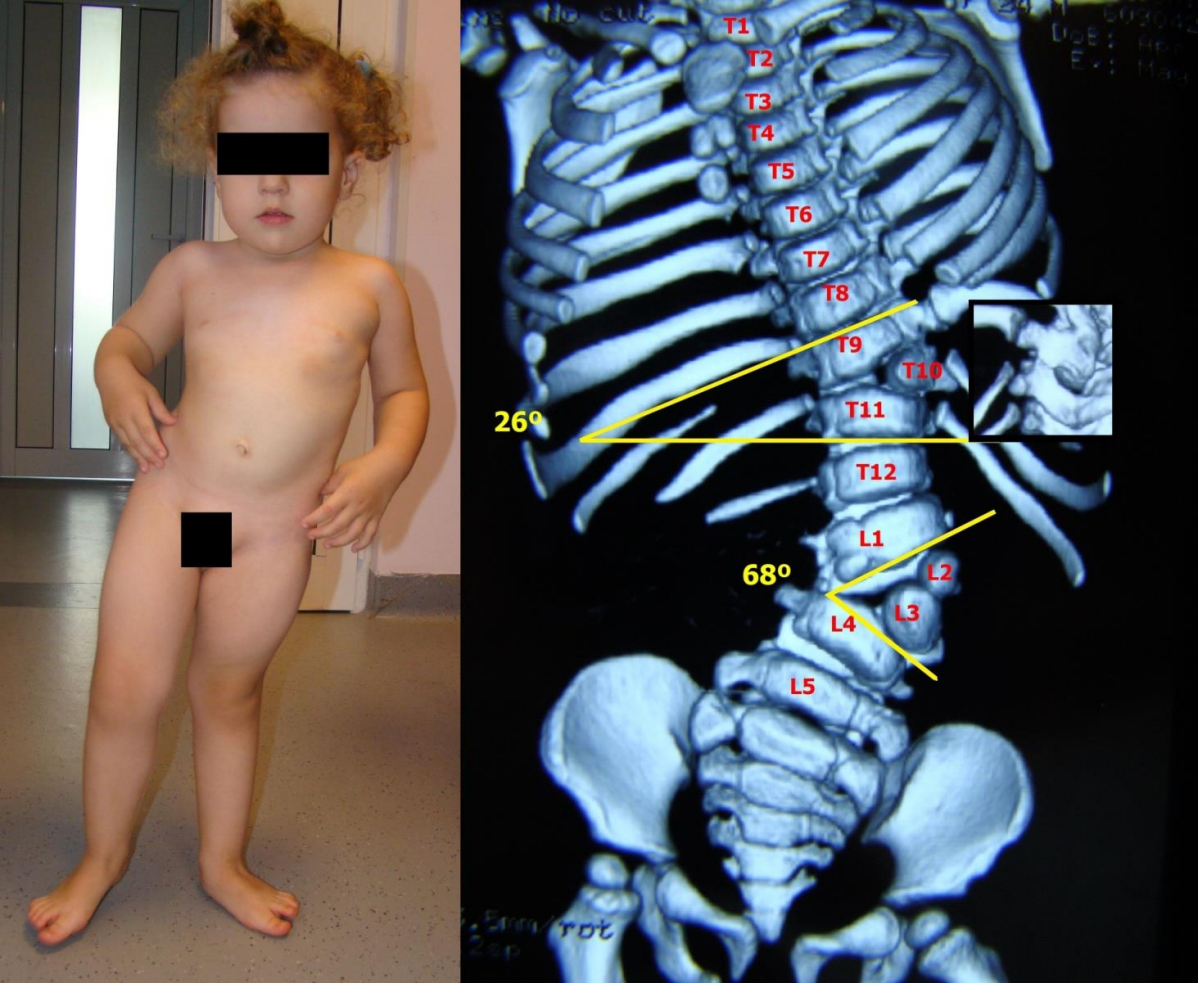
Congenital scoliosis with a high progression potential due to 3 hemivertebrae situated on the same side of the spine. Trunk shifting and shoulder imbalance are noticed at an early age

The treatment of congenital scoliosis is different from the adolescent idiopathic one. It is essential to assess the difference in growth of the concavity related to the convexity when choosing a particular procedure. The magnitude of the curve and the progressive rate are fundamental issues to the surgeon. Defects of segmentation usually induce a severe deformity. If the segmentation defect is associated to 2-3 fully segmented hemivertebrae, a maximal progression rate is present and significant curves are present at early ages (**Fig. 9**).

A severe curve in older children is very difficult to correct and a result is never obtained as in idiopathic scoliosis where correction may be up to 50-60%. In congenital scoliosis corrections of such kind of curves require laborious interventions, osteotomy or segmental resection, with high neurological risks [**33**] and low rates of success, not exceeding 20% as presented in different statistical data.

As a strategic aspect, the surgeon has to know that the preoperative planning has to identify the presence or absence of a dysraphic status [**34**] or syringomyelia. Always, the first aim is to stop the progression of the deformity.

**Acknowledgment.**

SG is part of POSDRU/159/1.5/S/137390
